# Caffeic acid phenethyl ester induces apoptosis in colorectal cancer cells via inhibition of survivin

**DOI:** 10.3906/biy-2003-18

**Published:** 2020-10-13

**Authors:** Ceren SARI, Ceren SÜMER, Figen CELEP EYÜPOĞLU

**Affiliations:** 1 Department of Medical Biology, Institute of Health Sciences, Karadeniz Technical University, Trabzon Turkey; 2 Department of Medical Biology, Faculty of Medicine, Karadeniz Technical University, Trabzon Turkey

**Keywords:** Anticarcinogenic agents, apoptosis, caffeic acids, colorectal cancer

## Abstract

Colorectal cancer is one of the most common types of cancer. Drug resistance and drug-induced damage of healthy tissues are major obstacles in cancer treatment. Therefore, to develop efficient anticancer therapy, it is necessary to find compounds that affect tumor cells, but do not exhibit toxicity to healthy cells. Caffeic acid phenethyl ester (CAPE) has been demonstrated to have anticancer properties in many types of cancer. In this study, the cytotoxic and apoptotic effects of CAPE on the RKO colorectal cancer cell line and CCD 841-CoN normal colorectal cell line was investigated. In addition, changes in the survivin expression were determined. According to the results, CAPE decreased cell viability in the RKO cell line in a dose-dependent manner. Likewise, CAPE induced apoptotic cell death in approximately 40% of the RKO cells. Furthermore, CAPE treatment increased the Serine 15 (Ser15) and Serine 46 (Ser46) phosphorylation of p53, while decreased the survivin expression. The results suggested that CAPE induced apoptosis by regulating p53 phosphorylation, leading to inhibition of the survivin expression. In accordance with the results, it is suggested that CAPE might be evaluated as an alternative drug in cancer therapy and further investigation is needed within this scope.

## 1. Introduction

Cancer is a heterogeneous group of diseases characterized by excessive cell proliferation or the disruption of apoptosis due to a loss of balance between cell division and cell death (Wong, 2011). Recently, cancer has been reported to be the second cause of death worldwide, following cardiovascular diseases (Global Burden of Disease Cancer et al. 2019). Among the different types of cancer, colorectal cancer is one of the most diagnosed and usually develops due to genetic and environmental factors (Kuipers et al. 2015). Over the last few years, many important roles in the molecular mechanism of colorectal cancer have been identified. However, these improvements have not fully contributed to diagnosis and treatment (Koehler et al., 2014). Although standard treatment methods, such as surgery, chemotherapy, and radiotherapy, have been used clinically, side effects of the chemotherapeutics and resistance to drugs are important problems that still have not been overcome (Urruticoechea et al., 2010). Therefore, alternative treatment strategies with high efficacy and few side effects are needed. In this context, various natural compounds that exhibit antiproliferative effects, by regulating apoptosis, are considered to be potential drug candidates in cancer therapy (Fulda, 2010).

Caffeic acid phenethyl ester (CAPE) is the main active phenolic compound of honeybee propolis. CAPE possesses different characteristics, such as antimicrobial, antiviral, antioxidant, and anticancer properties (Dos Santos and Monte-Alto-Costa, 2013; Sulaiman et al., 2014). It is known that CAPE is a candidate for anticancer drug studies due to its selective cytotoxic effects on cancer cells (Murtaza et al., 2014). Furthermore, CAPE treatment has been shown to negatively regulate cell proliferation via various signaling pathways and induce apoptosis upon the activation of p53 (Natarajan et al., 2013; Lin et al., 2015).

Survivin, a member of the inhibitor of apoptosis (IAP) protein family, is known to be a mitotic regulator that plays an important role in the abnormal proliferation of cancer cells. It is overexpressed in fetal tissues and many tumors, but barely present or completely absent in normal adult tissues. Overexpression of survivin is associated with decreased survival rate and it has been shown in many types of cancer, including colorectal cancer (Cheung et al., 2013). Survivin suppresses apoptosis by inhibiting caspases. Therefore, the downregulation of survivin blocks tumor growth by inducing apoptosis (Mita et al., 2008). Hence, targeting survivin in cancer treatment is thought to be a promising method.

p53 is a tumor suppressor gene and its mutations are frequently seen in tumorigenesis. Activated p53 is involved in different cellular mechanisms such as DNA repair, cell cycle arrest, and apoptosis (Hafner et al., 2019). p53 can regulate different physiological functions by being phosphorylated at multiple sites. Serine 15 (Ser15) phosphorylation plays the main role in DNA damage response. This phosphorylation impairs the interaction between p53 and its negative regulator, MDM2; hence, providing the dissociation of p53 from MDM2 and promoting the activation of p53. Ser15 phosphorylation also provokes a series of phosphorylation of p53, including Serine 46 (Ser46) phosphorylation, which further contributes to the induction of apoptosis. Ser46 phosphorylated p53 shows higher affinity to the promoters of apoptosis-related genes and activates apoptosis. Therefore, it is known that the phosphorylation of Ser46 is correlated with the apoptotic activity of p53 (Oda et al., 2000; Loughery et al., 2014).

In the molecular mechanisms of the disruption of apoptotic balance and the process of carcinogenesis, it is believed that there is a link between the regulatory functions of p53 and survivin. In cellular stress conditions, phosphorylated p53 inhibits antiapoptotic gene targets, including survivin. Moreover, it has been stated that survivin regulates p53 expression. p53-mediated apoptotic response has been shown to decrease via the overexpression of survivin (Mirza et al., 2002; Chen et al., 2016; Li et al., 2018). Since survivin suppresses apoptosis by inhibiting caspases, the downregulation of survivin is thought to be critical for successful p53-mediated apoptotic response and hence, cell death.

In the present study, the cytotoxic and apoptotic effects of CAPE treatment in colorectal cancer cells and normal colorectal cells was initially determined. After which, the protein levels of survivin, p53-Ser15, and p53-Ser46 were analyzed in order to gain insight into the molecular role of treatment with CAPE. Additionally, the alterations on the mRNA expression of survivin were verified. To conclude, the alternative drug potential of CAPE was evaluated in this study.

## 2. Materials and methods

### 2.1. Chemicals and reagents

CAPE, dimethyl sulfoxide (DMSO), acridine orange (AO), ethidium bromide (EB), and phosphatase inhibitors (Sigma Aldrich Corp., St. Louis, MO, USA); penicillin-streptomycin, phosphate buffered saline (PBS), fetal bovine serum (FBS), and McCoy’s 5A (modified) medium (Gibco, Paisley, UK); L-glutamine and trypsin-EDTA solution (Biological Industries, Kibbutz Beit Haemek, Israel); ethanol, beta mercaptoethanol, hydrochloric acid, methanol, sodium dodecyl sulfate (SDS), sodium hydroxide, and sodium chloride (Merck, Darmstadt, Germany); complete mini protease inhibitor (Roche, Basel, Switzerland); tween 20 (JT Baker, Phillipsburg, NJ, USA); bovine serum albumin (BSA) (Amresco, Solon, OH, USA); EMEM media (Lonza.Verviers, BE); ECL Plus western blot detection kit, antimouse-HRP (170-5047), and antirabbit-HRP(170-5045) antibodies (Bio-Rad Laboratories, Inc., Hercules, CA, USA); antisurvivin rabbit monoclonal antibody (ab76424), anti-p53 (phosphoS46) rabbit monoclonal antibody (ab76242), anti-p53 (phosphoS15) rabbit polyclonal antibody (ab1431), antiglyceraldehyde 3-phosphate dehydrogenase (GAPDH) mouse monoclonal antibody (ab125247), and WST-1 cell proliferation reagent kit (Abcam, Cambridge, MA, USA); RIPA lysis buffer (Santa Cruz Biotechnology, Inc., Dallas, TX, USA); BCA protein assay kit (Thermo Fisher Scientific Inc., Waltham, MA, USA); flow cytometric kits (BD Biosciences, San Diego, CA, USA); qRT-polymerase chain reaction (PCR) kits (QIAGEN, Hilden, Germany); and 5-fluorouracil (5-FU) (Ko **ç** ak Pharma, İstanbul, Turkey) were used in this study.

### 2.2. Cell culture

The RKO human colorectal carcinoma cell line (from ATCC, no.CRL-2577) and CCD 841-CoN human normal colorectal cell line (from ATCC, no.CRL-1790) were cultured in McCoy’s 5A and EMEM media, respectively. The media contained L-glutamine (2 µM/mL), 10% heat-inactivated FBS, and 1% penicillin and streptomycin. Cells were seeded in T75 cell culture flasks and incubated at 37 °C in a 5% CO_2_ humidified incubator. When the culture reached approximately 80% confluency, cells were removed from the flasks by trypsinization and passaged. This procedure was repeated until the end of the experiments.

### 2.3. Preparation of CAPE and 5-fluorouracil

0.1 g of CAPE was dissolved in 10 mL of DMSO. 5-FU in the ready-to-use format (0.05 g/mL) was used as a positive control. Further dilutions of CAPE and 5-FU were made in the culture media.

### 2.4. Cell viability

Cells were seeded at a density of 8 × 10^3^ cells per well in flat-bottomed 96-well cell culture plates and incubated for 18 h at 37 °C in a 5% CO_2_ humidified incubator. After incubation, the culture media was removed from the wells and the cells were washed with PBS. They were then treated with different concentrations of CAPE (0–100 µM) and 5-FU (0–40 µM) for the dose response studies. Culture media without CAPE or 5-FU were used for the untreated control group of cells. After treatment, the cells were incubated at 37 °C in a 5% CO_2_ humidified incubator for 72 h. Thereafter, 10 µL of WST-1 dye was added into each well and the cells were incubated for a further 4 h. Following the addition of WST-1, the cell viability was measured at a wavelength of 440 nm using a microplate reader (Versamax Molecular Devices, Sunnyvale, CA). Upon completion of the analysis of CAPE or 5-FU alone, the experiments were repeated using the effective doses of CAPE (75 µM) and 5-FU (10 µM) to perform the cotreatment study. Thus, the cells were seeded at a density of 8 × 10^3^ cells per well in 96-well cell culture plates and incubated for 18 h at 37 °C in a 5% CO_2_ humidified incubator. Following incubation, the culture media was removed and the cells were washed with PBS. Next, the cells were treated together with CAPE and 5-FU, and incubated at 37 °C in a 5% CO_2_ humidified incubator for 72 h. At the end of the incubation, the WST-1 assay was repeated as indicated.

### 2.5. Acridine orange/ethidium bromide staining

The cells were seeded at a density of 5 × 10^4^ cells per well in 6-well cell culture plates and incubated for 18 h at 37 °C in a 5% CO_2_ humidified incubator. After that, the cells were treated with 75 µM of CAPE in fresh media and incubated again at 37 °C in a 5% CO_2_ humidified incubator for 72 h. Following this incubation, the cells were harvested and suspended in PBS. A dye mixture of AO/EB that contained 5 µg/µL of AO and 3 µg/µL of EB was prepared. Next, 50 μL of the dye mixture was mixed gently with 50 µL of the cell suspension. After which, 20 µL of the mixed sample was transferred onto glass slides in the dark and covered with a coverslip. The morphology of the cells was examined immediately by fluorescence microscopy (Nikon, Minato, Tokyo, Japan).

### 2.6. Annexin V/7AAD apoptosis assay

The RKO and CCD 841-CoN cells were seeded in 6-well plates at a density of 2 × 105 and incubated for 18 h at 37 °C in a 5% CO_2_ humidified incubator. Next, the cell media was removed and the cells were treated with 75 µM of CAPE prior to 72 h of incubation. Afterwards, the cells were harvested and washed 3 times with PBS. Annexin V/7AAD staining was performed according to the manufacturer’s instructions (BD Biosciences, San Diego, CA, USA; Cat No: 559763). The cells were then analyzed using a flow cytometer (BD AccuriC6, Becton Dickinson, East Rutherford, NJ, USA).

### 2.7. Detection of caspase-3 activation

The cells were seeded in 6-well plates at a density of 2 × 10^5^ and incubated for 18 h at 37 °C in a 5% CO_2_ humidified incubator. After that, the cells were treated with 75 µM of CAPE in fresh media and incubated for 72 h. At the end of the incubation, the cells were harvested and washed 3 times with PBS. Active caspase-3 detection was performed using the PE Active Caspase-3 Apoptosis Kit according to the manufacturer’s instructions (BD Biosciences; Cat No: 550914). The cells were analyzed on a flow cytometer.

### 2.8. RNA isolation and real time quantitative PCR (qRT-PCR) analysis

Total RNA isolation was performed according to the instructions of the QIAGEN RNeasy Mini Kit. RNA was converted to complementary DNA (cDNA) using the QIAGEN QuantiTect Reverse Transcription Kit following isolation. The reaction mixture contained reverse transcriptase, buffer, primer mix, and template RNA. The PCR reaction was performed at 42 °C for 15 min and 95 °C for 3 min. Real-time quantitative PCR (qRT-PCR) was used to determine the expression level of the survivin. The forward and reverse primers of survivin comprised 5′-AGCCCTTTCTCAAGGACCAC-3′ and 5′-CTCTATGGGGTCGTCATCTGG-3′, respectively. β-actin was used as the standard control. The forward and reverse primers of β-actin comprised 5′-CTGGCACCACACCTTCTACAATG-3′ and 5′-CCTGGTAGATGGGCACAGTGTG-3′, respectively. The reaction was performed at 95 °C for 15 min (pre-incubation) for 1 cycle; 94 °C for 15 s, 60 °C for 30 s, and 72 °C for 30 s (amplification) for 40 cycles, and 40 °C for 30 s for 1 cycle (cooling) in a Roche Light Cycler 480-II System (Rotkreuz, Switzerland).

### 2.9. Western blot

The cells were seeded in 6-well plates at a density of 4 × 10^5^ and incubated for 18 h at 37 °C in a 5% CO_2_ humidified incubator. Afterwards, the cells were treated with 75 µM of CAPE in fresh media and incubated for 72 h. Following incubation, the cells were washed with ice-cold PBS at 4 °C. After that, 250 µL of RIPA lysis buffer, supplemented with protease and phosphatase inhibitors, were added to cells and shaken on an orbital shaker, on ice, for 30 min. Cell lysates were removed from the 6-well plates using a cell scraper and centrifuged at 13,000 *g* for 15 min at 4 °C. The protein concentration was determined using the BCA Protein Assay Kit. Equal amounts of protein were loaded on 12.5% SDS-polyacrylamide gel and electrophoresis was performed. The proteins were transferred to nitrocellulose membranes after electrophoresis. The membranes were blocked for 1 h at room temperature using 2.5% nonfat dried milk and 2.5% BSA in tris-buffered saline (TBS). Blotted membranes were incubated with primary antibodies at 4 °C overnight. The blocking solutions, comprised antisurvivin rabbit monoclonal antibody at a dilution of 1/2500, anti-p53 (phosphoS15) rabbit polyclonal antibody at a dilution of 1/250, anti-p53 (phosphoS46) rabbit monoclonal antibody at a dilution of 1/1000, and anti-GAPDH mouse monoclonal antibody at a dilution of 1/5000. Following the primary antibody incubations, the membranes were washed 3 times with TBS with tween, containing 0.5% tween 20. Afterwards, the membranes were incubated with antimouse-HRP and antirabbit-HRP secondary antibodies for 1 h at room temperature. Thereafter, the membranes were developed in the dark using the ECL Plus Western blotting detection kit. In the last step, the membranes were visualized and analyzed using the ChemiDoc MP Imaging system (BioRad, Hercules, CA, USA).

### 2.10. Statistical analysis

Statistical data analysis was performed using GraphPad Prism (Version 8.0; La Jolla, USA). All of the experiments were repeated at least 3 times with multiple replicates based on the experiment. Differences between the untreated and CAPE or 5-FU treatment groups were analyzed using the student t-test. Differences between the untreated control, CAPE treatment, 5-FU treatment, and CAPE +5-FU treatment groups were compared using one-way ANOVA analysis with the Tukey multiple comparisons test. All of the data were expressed as the mean ± SD and P < 0.05 was considered as statistically significant.

## 3. Results

### 3.1. Effects of CAPE on the cell viability of the RKO cells

**Figure 1:**
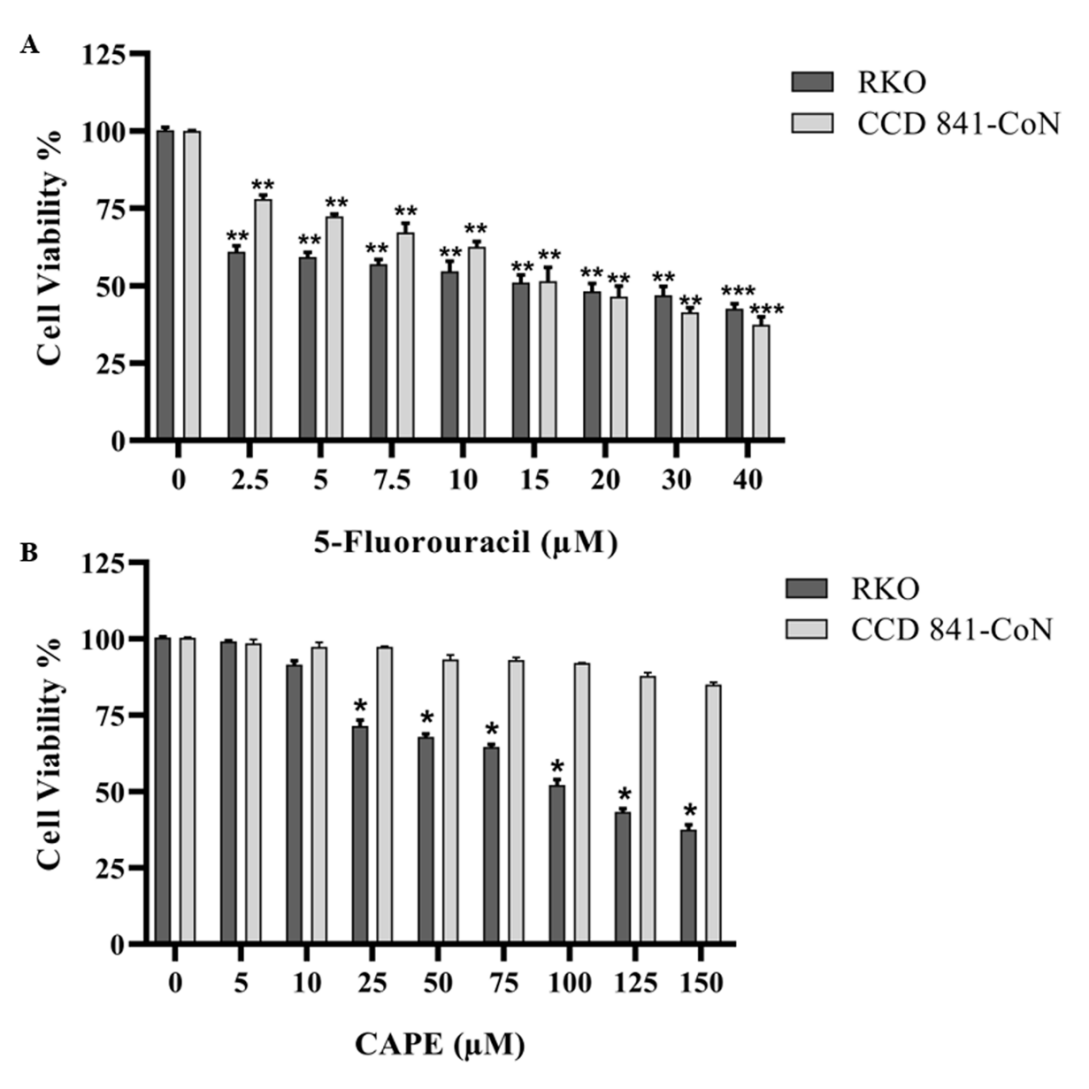
Concentration-dependent change of the RKO and CCD 841-CoN cell viabilities following 5-FU treatment (A) or CAPE treatment (B). Statistical significance between the negative controls and treated groups was compared using the student t-test. *P < 0.05, **P < 0.01.

**Figure 2:**
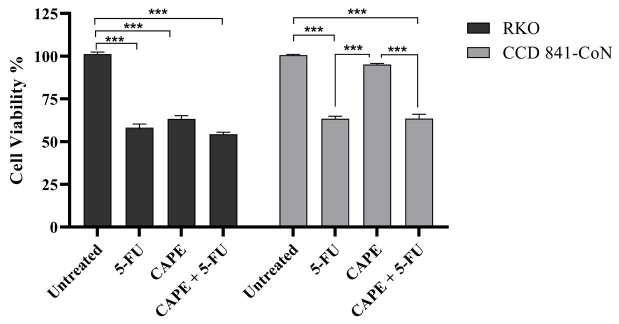
Survival rates of the RKO and CCD 841-CoN cells following treatment with effective doses of 5-FU, CAPE, or the combination of CAPE +5-FU. Statistical significance between the untreated control group, CAPE treatment group, 5-FU treatment group, and CAPE +5-FU treatment group was compared using one-way ANOVA analysis and the Tukey multiple comparisons test. ***P < 0.001.

The RKO and CCD 841-CoN cells were treated with various concentrations of CAPE and 5-FU. Cell viability was determined using the WST-1 assay. The cytotoxic effects of 5-FU (2.5–40 µM) increased in a dose-dependent manner in both cell lines (Figure 1a). CAPE treatment (5–150 µM) caused an increased cytotoxic effect in the RKO cells in a dose-dependent manner, while the same effect was not observed in the normal CCD 841-CoN cells (Figure 1b). The IC50 values of CAPE were 108 µM in the RKO cells and 446.5 µM in the CCD 841-CoN cells. The IC50 values obtained for 5-FU were 23.5 µM in the RKO cells and 24.2 µM in the CCD 841-CoN cells. The dilutions of CAPE (75 µM) and 5-FU (10 µM) that killed nearly 40% of the cells were used to observe whether they would have any combined effect at the selected doses. Cotreatment of CAPE with 5-FU caused approximately a 10% increase in the cytotoxicity of the RKO cells when compared to the CAPE treatment alone, while a 5% increase was determined when compared to the 5-FU treatment alone. On the other hand, the CCD 841-CoN cells were highly affected by the CAPE +5-FU combination due to the presence of 5-FU in the combined drug (Figure 2). To clearly observe the effect of the CAPE treatment at the mRNA and protein expression levels in subsequent experiments, it was decided to use a dose of 75 µM of CAPE, which caused significant cytotoxic activity in the RKO cell line, but was ineffective in the CCD 841-CoN cell line.

### 3.2. Effects of CAPE treatment on apoptotic cell morphology of the RKO cells

**Figure 3:**
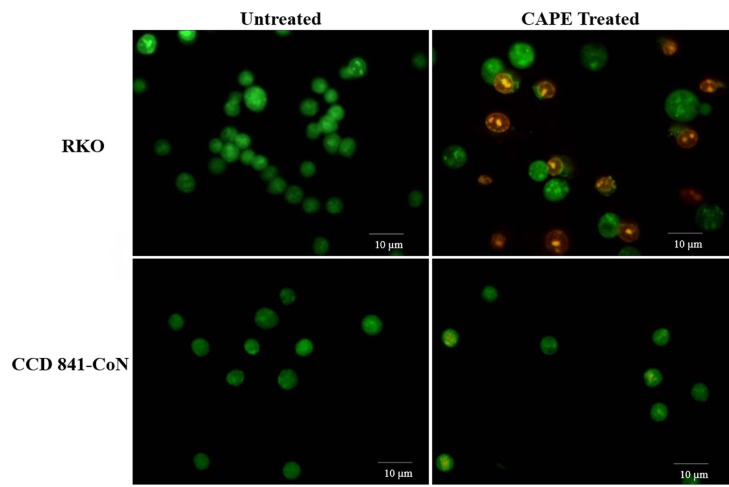
Fluorescence imaging of cell death in the RKO and CCD 841-CoN cells after CAPE treatment. Magnification 200×.

To observe the changes in the cell morphology, the RKO and CCD 841-CoN cells were treated with 75 µM of CAPE for 72 h and then stained with AO/EB. It was observed that AO stained all of the cells, while EB permeated only the dead or dying cells (Baskićet al., 2006). Therefore, the green-stained nuclei-containing cells were living cells that were only stained with AO, while the orange-stained nuclei-containing cells were apoptotic cells that were permeable to both AO and EB. According to fluorescence microscope results, the RKO cells showed apoptotic cell morphology after the CAPE treatment. No morphological change was observed in the normal CCD 841-CoN cells (Figure 3).

### 3.3. Effects of CAPE treatment on apoptotic cell population and caspase-3 activation of the RKO cells

**Figure 4:**
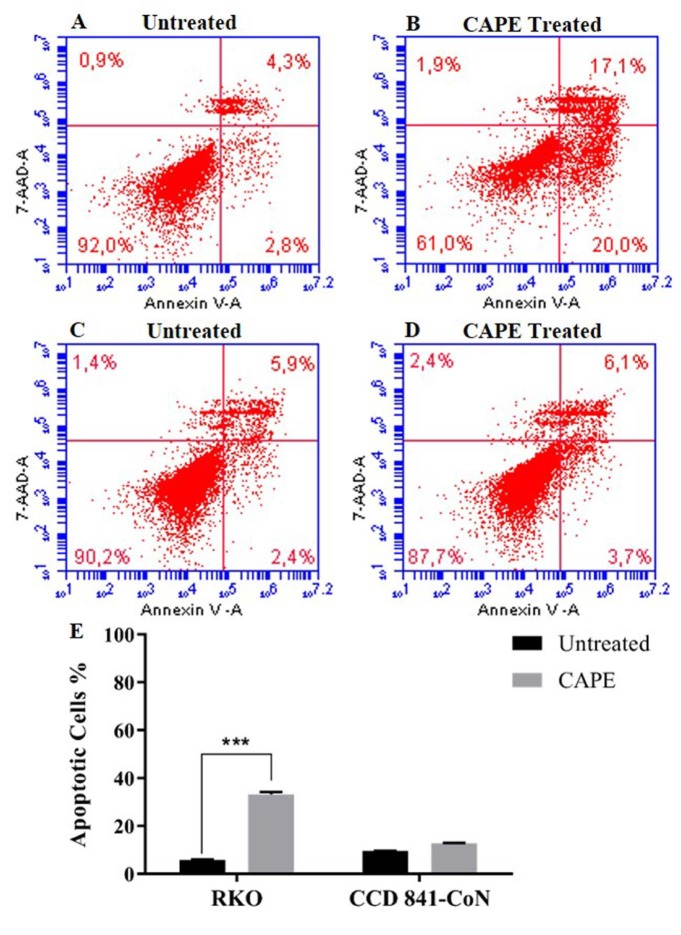
Flow cytometric analysis of the Annexin V/7AAD staining following CAPE treatment in the RKO (A, B) and CCD 841-CoN (C, D) cells. Statistical significance between the negative controls and treated groups was compared using the student t-test (E). ***P < 0.001.

**Figure 5:**
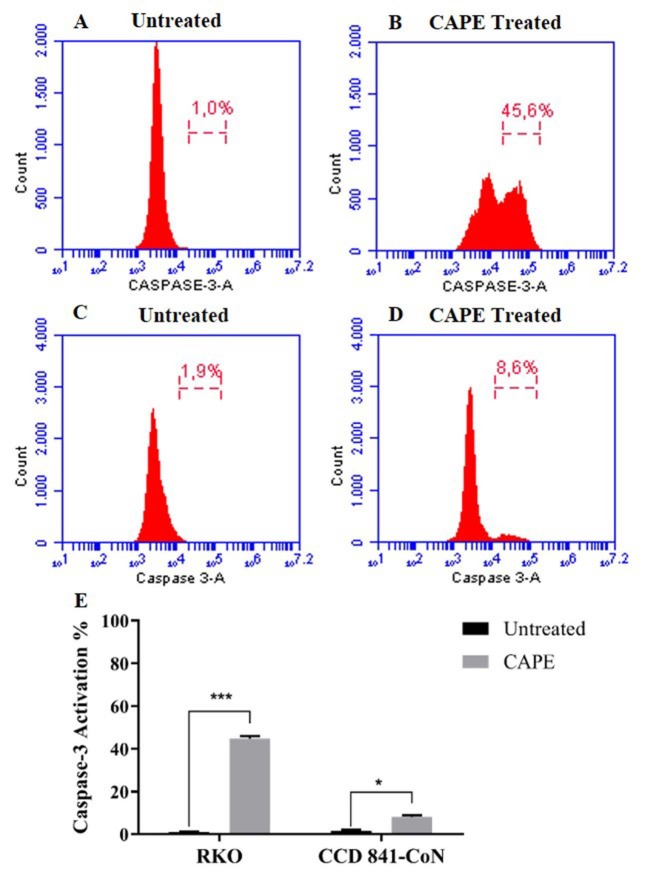
Flow cytometric analysis of the caspase-3 activity following CAPE treatment in the RKO (A, B) and CCD 841-CoN (C, D) cells. Statistical significance between negative controls and treated groups was compared using the student t-test (E). *P < 0.05, ***P < 0.001.

The apoptotic cell population and caspase-3 activation caused by the CAPE treatment in RKO and CCD 841-CoN cells were investigated using flow cytometry. A high proportion of the apoptotic cell population was detected in the RKO cells that were treated with 75 µM of CAPE for 72 h when compared to the untreated cells (Figures 4a and 4b). In the CCD 841-CoN cells, there was no change in the apoptotic cell population following the CAPE treatment (Figures 4c–4e). For the caspase-3 activity analysis, data from the untreated cells were considered as basal caspase activity. It was found that the caspase-3 activity increased by approximately 45% in the RKO cells and 8% in the CCD 841-CoN cells after the CAPE treatment (Figure 5).

### 3.4. Survivin gene expression following CAPE treatment

**Figure 6:**
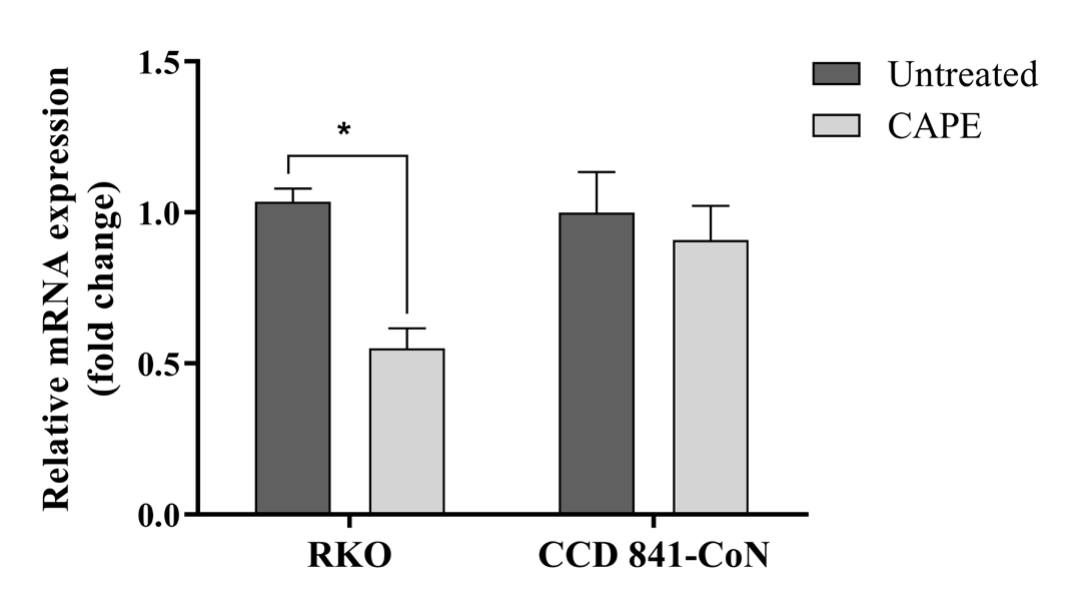
Relative mRNA expression levels of survivin in the RKO and CCD 841-CoN cells following CAPE treatment. Statistical significance between the negative controls and treated cell groups was compared using the student t-test. *P < 0.05.

qRT-PCR was performed to examine changes in the survivin gene expression in the RKO and CCD 841-CoN cell lines. CAPE treatment of the RKO cells resulted in a 50% reduction in the mRNA expression level of survivin. No statistically significant change was observed in the survivin mRNA expression level of the CCD 841-CoN cell line (Figure 6).

### 3.5. Protein levels of survivin, phosphorylated p53-Ser15, and phosphorylated p53-Ser46 after CAPE treatment

**Figure 7:**
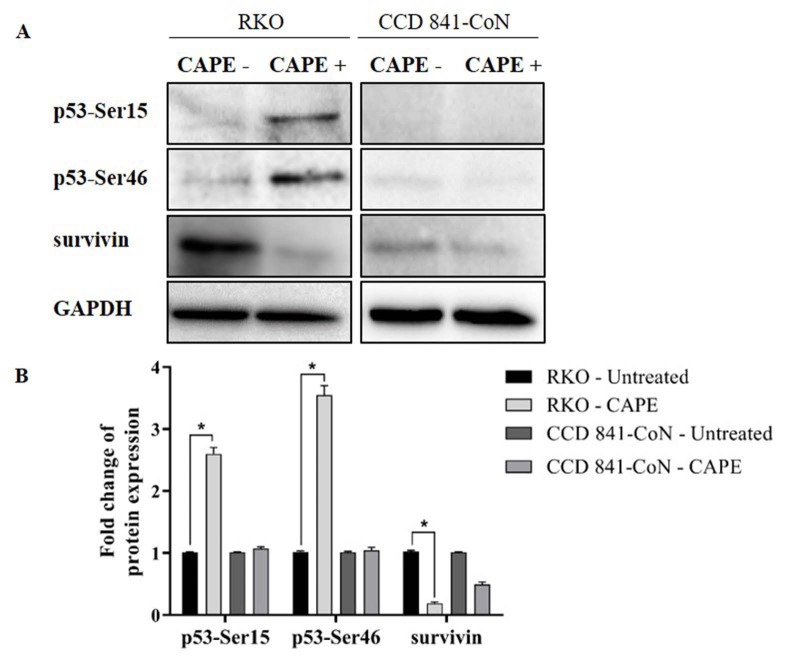
Effect of CAPE treatment on p53-Ser15, p53-Ser46, and survivin protein levels of the RKO and CCD 841-CoN cells (A). Expressions of p53-Ser15, p53-Ser46, and survivin were normalized to GAPDH. Fold changes were calculated relative to the untreated groups using the student t-test (B). *P < 0.05.

The expression levels of survivin, and proteins p53-Ser15 and p53-Ser46 in the RKO and CCD 841-CoN cells were examined via western blot analysis. Consistent with the results of the qRT-PCR, the CAPE treatment decreased the survivin protein level by approximately 5-fold in the RKO cells. Moreover, the phosphorylated p53-Ser15 level increased by 2.6 fold, while the p53-Ser46 protein level increased by 3.5 fold in the RKO cells following CAPE treatment. There was no significant change in the p53-Ser15 and p53-Ser46 expression levels in the CCD 841-CoN cells (Figure 7).

## 4. Discussion

Cancer is the second leading cause of death worldwide. Aggressive progression and drug resistance have become a challenge in the treatment of cancer. Recent studies on cancer therapeutics have focused on targeting cellular structures, such as survivin, which is involved in apoptotic processes. Survivin is an IAP protein that is highly expressed in most types of cancer (Altieri, 2003; Peery et al., 2017). It is known that survivin is involved in tumorigenesis by inhibiting caspases, such as caspase-3, -7, and -9, and blocking p53-mediated apoptosis (Temrazet al., 2013; Chenet al., 2016). Increased invasion and metastasis have been shown to be related with an overexpression of survivin in colorectal carcinoma (Chu et al., 2012). Furthermore, survivin has been associated with reduced survival rates of colorectal cancer patients (Kawasaki et al., 1998). Different studies have shown that the expression of survivin could be considered as a prognostic marker. Furthermore, high expression levels of survivin in cancer cells and its correlation with chemotherapy resistance have suggested that it may be one of the key structures to target for alternative treatment methods (Shenet al., 2009). Therefore, the inhibition of survivin is thought to be a promising therapeutic strategy in cancer treatment (Peeryet al., 2017).

In alternative treatment-oriented cancer studies, anticancer agents that selectively target cancer cells, rather than healthy cells, were selected for use. CAPE is a well-known component of propolis and has been widely used in anticancer studies. Many reports have stated that CAPE could induce apoptosis in different types of cancer cells. Moreover, CAPE has been demonstrated to selectively kill cancer cells (He et al. 2014; Murtaza et al. 2014). Therefore, it was aimed herein to investigate the anticancer effects of CAPE on RKO colorectal cancer cells and determine changes in the survivin expression following CAPE treatment.

Previous studies have shown that CAPE has the ability to inhibit cell proliferation and tumor growth (Chen et al., 2004; Kabala-Dzik et al., 2018). Therefore, in vitro cell viability experiments were performed in this study to analyze the effects of CAPE alone or in combination with 5-FU. It was found that CAPE and 5-FU exhibited cytotoxic effects on colorectal cancer cells in a dose-dependent manner. However, the healthy colorectal cells were not affected by the CAPE treatment, while 5-FU significantly decreased the cell viability of the healthy cells (Figures 1 and 2). Therefore, the selective cytotoxic effects of CAPE on the cancer cells were determined, as previously mentioned in the literature (Murtaza et al., 2014). Consistently, the cancer cells started to exhibit apoptotic morphology following the CAPE treatment, as observed via the AO/EB staining (Figure 3). When the apoptotic activity of CAPE was further analyzed, increased caspase-3 activity and apoptotic population was detected in the RKO cells treated with CAPE (Figures 4 and 5). CAPE has been shown to increase caspase-3 activity in different cell lines and consequently induce apoptosis (Dziedzicet al., 2017; Kabala-Dzik et al., 2018). In addition to these studies, the data herein indicated that CAPE induced apoptosis by increasing caspase-3 activity in the RKO colorectal cancer cells, while low levels of caspase-3 activation occurred in the CCD 841-CoN cells treated with CAPE. 

Previous research has reported that CAPE treatment alters the expression of different apoptotic genes in cancer cells (Lin et al., 2012; Gherman et al., 2016). In this study, it was detected that the CAPE treatment significantly reduced the mRNA expression level of survivin when compared to the untreated group of cells (Figure 6). It is important to note that CAPE treatment could reduce the level of mRNA expression in survivin, which is known to be highly expressed in cancer cells and is associated with resistance in treatment.

Following the detection of a change in the mRNA level of survivin, the effects of the CAPE treatment on the protein levels of survivin, p53-Ser15, and p53-Ser46 were observed. Post-translational modification of p53 is known to contribute to the regulation of p53 stabilization and function. Specifically, the phosphorylation of p53 at different residues determines its transcriptional activity, thus causing specific types of responses to cellular stress. Ser15 phosphorylation of p53 characterizes an early cellular response to DNA damage and Ser46 is an important phosphorylation site for the induction of subsequent responses, particularly apoptosis (Kurihara et al., 2007). In response to cellular stress following DNA damage, phosphorylated p53 binds DNA and activates apoptotic genes or suppresses antiapoptotic gene targets, including survivin. On the other hand, survivin has been reported to regulate p53 expression. The overexpression of survivin reduces p53-mediated apoptotic response and this suggests that the inhibition of survivin may play an important role in p53-mediated apoptosis (Mirza et al., 2002; Chen et al., 2016; Li et al., 2018). In the current study, a decrease in the protein expression of survivin was detected following CAPE treatment. Conversely, the Ser46 and Ser15 phosphorylation of p53 increased (Figure 7). These findings indicated that CAPE treatment can lead to p53 activation by Ser15 phosphorylation, which in turn may trigger the activation of apoptotic genes by Ser46 phosphorylation, thereby resulting in the suppression of survivin expression.

Taken together, it was concluded that CAPE selectively increased apoptotic activity through p53 activation and survivin inhibition in colorectal cancer cells. Since this apoptotic activity occurred selectively, without damaging healthy cells, and survivin was the target of the CAPE treatment, it is suggested that CAPE has the potential to be an alternative anticancer agent. In this context, understanding the mechanism of apoptosis, which was induced by CAPE through p53 and survivin, might be helpful to design alternative therapeutic strategies for the treatment of colorectal cancer. To better understand this, analyzing the changes in the levels of different proapoptotic and antiapoptotic proteins, such as Bax, PUMA, NOXA, Bcl-2, and XIAP, following CAPE treatment will contribute to the study. Furthermore, expanding this study by using a known survivin inhibitor, such as sepantronium bromide (YM155), or by silencing the survivin and p53 expression via knockdown or knockout studies, will provide substantial information about the effects of CAPE treatment. Thereafter, in vivo studies using animal models will help to describe the biological activities of CAPE. Thus, further studies are required to evaluate the detailed mechanisms of action.
